# Changes in glomerular filtration rate in patients with body mass index ≥35 kg/m^2^ treated with metabolic and bariatric surgery versus GLP‐1 agonist at 1‐year follow‐up

**DOI:** 10.1002/osp4.782

**Published:** 2024-08-10

**Authors:** Diana Cristina Henao‐Carrillo, Mayra Alejandra Jurado‐Florez, Óscar Mauricio Muñoz

**Affiliations:** ^1^ Pontificia Universidad Javeriana Bogotá Colombia; ^2^ Endocrinology Unit Hospital Universitario San Ignacio Bogotá Colombia; ^3^ Department of Internal Medicine Hospital Universitario San Ignacio Bogotá Colombia

**Keywords:** glomerular filtration rate, GLP1 receptor analogs, metabolic and bariatric surgery, obesity

## Abstract

**Background:**

Metabolic and bariatric surgery (MBS) reduces glomerular hyperfiltration. The renoprotective effects of GLP‐1 analogs were derived from clinical studies in type 2 diabetes (T2D). The objective of this study was to evaluate the changes in glomerular filtration rate (GFR) over time associated with weight loss in patients with a BMI ≥ 35 kg/m^2^ treated with liraglutide compared with patients treated with MBS.

**Methods:**

A longitudinal study derived from a retrospective cohort of patients with BMI ≥ 35 kg/m^2^ treated with either MBS or liraglutide 3 mg/day, with follow‐up ≥1 year. Clinical variables, baseline GFR, and 1‐year GFR were analyzed. A generalized estimating equation (GEE) model was used to compare changes in GFR between both groups while controlling for confounding variables.

**Results:**

A total of 159 patients were included in the analysis. Of these, 129 patients underwent MBS (median age 60.5 years [IQR 51.8–66.6], body mass index (BMI) 40.9 kg/m2 [IQR 0.68–0.89]), and 30 patients were treated with liraglutide (median age 56 years [IQR 46–62], BMI 37.4 kg/m^2^ [IQR 0.69–0.93]). No difference in baseline GFR or at 12 months of follow‐up was found between the two interventions. GEE analysis revealed an increase of 0.32 mL/min/1.73 m^2^ per month of follow‐up. Factors associated with a greater increase in GFR were the percentage total weight loss (%TWL) (0.12 mL/min/1.73 m^2^, *p* = 0.023) and baseline GFR (0.69 mL/min/1.73 m^2^, *p* > 0.001) for both interventions, independent of a history of T2D.

**Conclusion:**

In patients with BMI ≥ 35 kg/m^2^, changes in GFR are related to %TWL and baseline GFR, regardless of the presence of diabetes or the type of intervention used.

## Graphical abstract

Evidence regarding changes in glomerular filtration rate (GFR) over time in patients with a body mass index (BMI) ≥35 kg/m^2^ treated with liraglutide 3 mg/day is limited. In patients with BMI ≥ 35 kg/m^2^, changes in GFR are related to %TWL and baseline GFR, regardless of the presence of diabetes or the type of intervention used (metabolic and bariatric surgery or liraglutide).

**Abbreviations**
%TWL: percentage total weight loss
AR‐GLP1: GLP1 receptor analogs
BMI: body mass index
GEE: generalized estimating equations
GFR: glomerular filtration rate
IQR: interquartile range
MBS: metabolic and bariatric surgery
SD: standard deviation
T2D: type 2 diabetes mellitus

## 1 INTRODUCTION

Obesity is acknowledged as an independent factor contributing to the onset of chronic kidney disease (CKD).^1^ Excessive body weight induces glomerular hyperfiltration, resulting in albuminuria and progressive decline in glomerular filtration rate (GFR) due to obesity‐related specific glomerular injury, particularly focal and secondary segmental glomerulosclerosis.^2^ In addition, obesity accelerates the decline in kidney function in individuals with CKD not related to obesity, such as those with glomerulonephritis. It is estimated that individuals with a body mass index (BMI) greater than 35 kg/m^2^ have up to a 3.4‐fold increased risk of developing CKD.^1^

Reducing BMI improves obesity‐related kidney damage, including GFR,^3^, ^4^ and it has been observed that for every kilogram of weight lost, there is a 4% reduction in albuminuria and proteinuria.^5^ Several studies in patients treated with Metabolic and bariatric surgery (MBS) have demonstrated an improvement in GFR after surgery, with a reduction in glomerular hyperfiltration or an increase in GFR by up to 13 mL/min (*p* < 0.0001).^6^, ^7^ While some studies associate the improvement in renal function to the effect of weight loss on the remission of diabetes and hypertension, recent data suggest that there is no significant difference in the remission of these comorbidities and the improvement in GFR. Therefore, the sole act of reducing weight is sufficient to enhance renal function.^3^ Consequently, MBS is considered a strategy to treat, halt, or even prevent the development of CKD in patients with severe obesity.^8^ However, there are currently no clinical practice guidelines for the management of patients with obesity‐related kidney disease.^9^

In recent years, pharmacological therapies with glucagon‐like peptide‐1 receptor analogs (GLP‐1 RAs) have been approved, resulting in significant weight loss of up to 20% of baseline weight^10^ in patients with obesity. This has positioned them as an alternative for managing this condition. In large prospective studies in people with type 2 diabetes (T2D), GLP‐1 RAs have not yet demonstrated a significant improvement in renal outcomes beyond a reduction in albumin excretion. Post hoc analyses of cardiovascular safety trials such as LEADER (liraglutide),^11^ SUSTAIN‐6 (semaglutide),^12^ and REWIND (dulaglutide)^13^ have reported modest reductions in the rate of decline in GFR, particularly notable in individuals with GFR <60 mL/min/1.73 m^2^. One real‐world study reported a reduction in the rate of major renal events (renal replacement therapy, renal death, and hospitalization for renal events). However, these studies used lower doses of GLP‐1 RA than those used in the treatment of obesity, where greater weight loss occurs.^14^

Evidence regarding changes in GFR over time in patients with a BMI ≥35 kg/m2 treated with liraglutide 3 mg/day is limited. The objective of this study was to evaluate changes in GFR over time associated with weight loss in patients with a BMI ≥35 kg/m2 treated with liraglutide 3 mg/day compared with those observed in patients treated with MBS.

## 2 METHODS

A longitudinal study was conducted based on a retrospective cohort of patients managed at the Obesity Clinic of the Hospital Universitario San Ignacio, a reference hospital in Bogotá (Colombia), from January 2011 to December 2021. All patients aged 18 years and older with a BMI greater than or equal to ≥35 kg/m2 who underwent any type of MBS or were treated with liraglutide 3.0 mg and had a follow‐up of at least 6 months were included.

Patients with secondary MBS (reintervention), those who underwent surgery at another institution, with an estimated GFR at the time of surgery <30 mL/min/1.73 m^2^, advanced Child–Pugh class C cirrhosis, active neoplasia, pregnancy, chronic use of non‐steroidal anti‐inflammatory drugs, or those with medical conditions that adversely affect renal function unrelated to metabolic disease (rheumatologic diseases, genetic disorders, toxin‐related kidney disease, or a history of acute kidney injury in the three months prior to surgery) were excluded. The study was reviewed and approved by the institutional ethics committee (171–2022). The Obesity Clinic Program includes serial follow‐up visits scheduled at least every 3 months during the first‐year post‐intervention. Data were collected retrospectively from systematically collected medical records. Variables included were date of surgery, date of initiation of GLP‐1 analogs, baseline and serial creatinine levels, baseline and serial GFR, comorbidities (hypertension, diabetes, and obstructive sleep apnea), pre‐operative and serial weight, initial and serial BMI, as well as the percentage total weight loss (%TWL). %TWL was defined as baseline weight minus post‐intervention weight divided by baseline weight multiplied by 100. GFR was calculated using the CKD‐EPI 2021 formula.^15^

Continuous variables were reported as mean and standard deviation or median and interquartile range, depending on the distribution of the variables. The Shapiro‐Wilk test was used to assess the normality assumption. Categorical variables were reported as frequencies and percentages. To estimate the trend over time of the estimated GFR using CKD‐EPI, a longitudinal analysis was performed using generalized estimating equation (GEE). This approach took into account the autocorrelation of repeated observations within the same patient and allowed us to evaluate how the average of the response variables changed as a function of each of the covariates studied. An interchangeable correlation structure was used. A multivariable GEE was employed to identify the coefficients of each covariate. The time model with a significant contribution (*p*‐value <0.05) and the lowest quasi‐likelihood under the independence model criterion (QIC) represented the best fit for the data. For sensitivity analysis, GEE models were also adjusted assuming an unstructured or “independent” correlation structure, with no significant changes in the results. Statistical analysis was performed using STATA® software (Stata Statistical Software: Release 17. College Station, Texas: StataCorp LLC).

## 3 RESULTS

A total of 159 patients were included, of whom 129 underwent MBS and 30 received medical management with liraglutide 3.0 mg. The mean age was 60.5 (IQR 51.8–66.6) and 56 (IQR 46–62) years for each group. BMI [40.9 (37.7–45.0) versus 37.4 (36.0–40.5), *p* < 0.001] and the presence of obstructive sleep apnea (72.1% vs. 68.8%, *p* < 0.001) were higher in the surgically managed group; however, baseline creatinine, GFR, glycated hemoglobin levels, and the presence of arterial hypertension and diabetes were similar between the groups (Table 1).

**TABLE 1:** Baseline characteristics of patients.

Variable	MBS *n* = 129	Liraglutide *n* = 30	*p*‐value
Age in years, median (IQR)	60.5 (51.8–66.6)	56 (46.2–62.5)	0.023
Female sex, *n* (%)	107 (82.9)	21 (70.0)	0.190
BMI kg/*m* ^2^, median (IQR)	40.9 (37.7–45.0)	37.4 (36.0–40.5)	<0.001
Baseline creatinine, median (IQR)	0.78 (0.7–0.9)	0.79 (0.7–0.9)	0.371
Hypertension, *n* (%)	68 (52.7)	61 (47.3)	0.951
HbA1C %, median (IQR)	5.97 (5.5–6.6)	5.8 (5.4–6.5)	0.860
Diabetes, *n* (%)	53 (41.0)	10 (33.3)	0.434
Obstructive sleep apnea, *n* (%)	93 (72.1)	11 (68.8)	<0.001
Mild (AHI, 5–14.9 events per hour)	26 (20.1)	11 (68.8)	
Moderate (AHI, 15–29.9 events per hour)	18 (13.9)	0 (0)	
Severe (AHI, ≥30 events per hour)	49 (38.0)	0 (0)	
GFR^a^ basal	90.6 (79.1–100.3)	95.3 (79.8–108.4)	0.280
30–60 mL/min/1,73 m^2^	5 (3.8)	2 (6.7)	
61–89 mL/min/1,73 m^2^	55 (42.6)	11 (36.6)	
≥90 mL/min/1,73 m^2^	69 (53.5)	17 (56.6)	

Abbreviations: AHI, apnea‐hypopnea index; BMI, body mass index; GFR, glomerular filtration rate; HbA1c, glycated hemoglobin A1c; IQR, interquartile range; MBS, metabolic and bariatric surgery.

^a^ Calculated by CKD‐EPI 2021.

**FIGURE 1:**
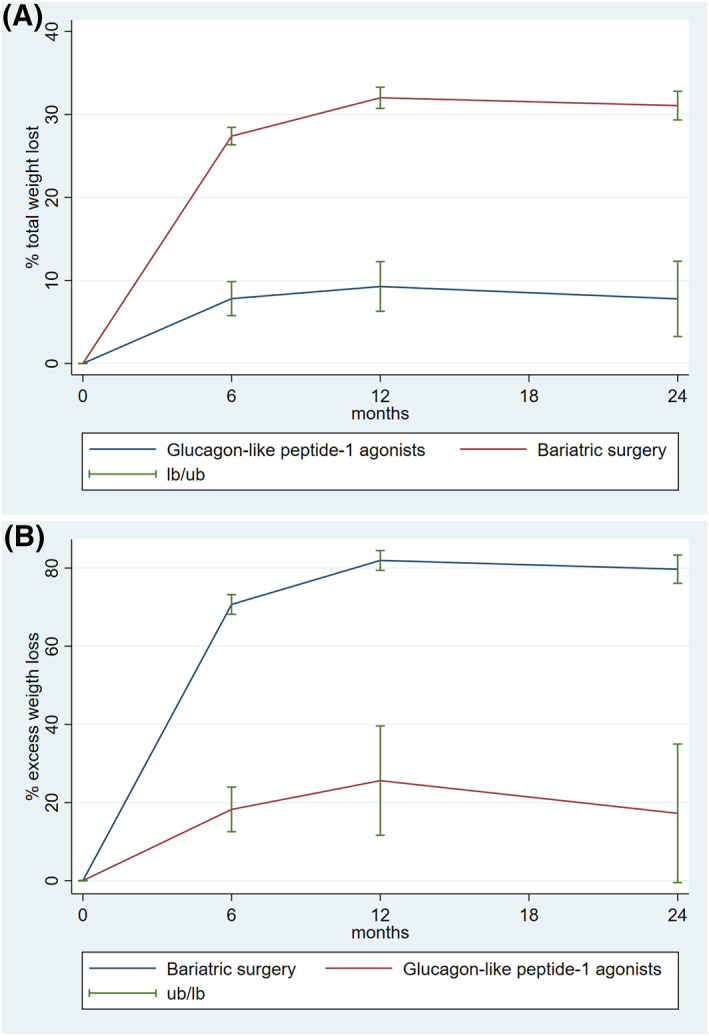
(A) Weight change as a percentage of total weight loss between patients undergoing bariatric surgery and those treated with GLP‐1 agonists during the 24‐month follow‐up period. (B) Weight change as a percentage of excess weight loss between patients undergoing bariatric surgery and those treated with GLP‐1 agonists during the 24‐months follow‐up period.

A statistically significant difference in %TWL was observed in favor of those who underwent surgical treatment (Table 2). It peaked within the first 12 months after surgery and then plateaued over time, regardless of whether surgical or medical treatment was used (Figure 1).

**TABLE 2:** Total percentage of weight lost in patients treated with AR‐GLP1 and MBS during 2 years of follow‐up.

Time (months)	Liraglutide	MBS	*p*‐value
	%TWL, median (IQR)	%TWL, median (IQR)	
6	7.6 (5.2–11)	27.2 (24.3–31.2)	<0.001
12	9.3 (5.3–13.3)	31.8 (27.7–36.2)	<0.001
24	7.2 (0–11.3)	30.9 (26.7–36.4)	<0.001

Abbreviations: %TWL, percentage of total weight loss; IQR, interquartile range, MBS, metabolic and bariatric surgery.

**FIGURE 2:**
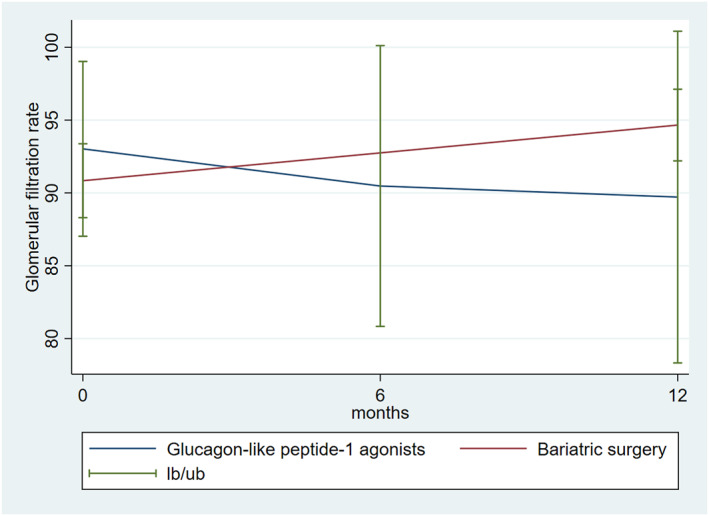
Changes in glomerular filtration rate between patients undergoing bariatric surgery and those treated with GLP‐1 agonists during the 12‐months follow‐up period.

Changes in GFR were compared between patients treated with medical and surgical management. No statistically significant differences were found at 1 year of follow‐up (Table 3) (Figure 2).

**TABLE 3:** Change in glomerular filtration rate over 1 year in patients treated with AR‐GLP1 and MBS.

Time	Liraglutide		MBS		*p*‐value
	eGFR	(IQR)	eGFR	(IQR)	
Initial, median (IQR)	94.6	(80–108)	92	(80.3–110)	0.498
12 months, median (IQR)	91	(66–103)	96.5	(86–105)	0.425

Abbreviations: IQR, interquartile range; eGFR, estimated glomerular filtration rate calculated by CKD‐EPI 2021; MBS, metabolic and bariatric surgery.

The GEE model allowed analysis of different patterns of changes in GFR over time. Longitudinal analysis showed an increase of 0.32 (95 IC 0.13–0.52, *p* < 0.001) mL/min/1.73 m^2^ per month of follow‐up, controlling for risk factors such as baseline weight, age, diabetes, arterial hypertension, baseline GFR, and %TWL. Factors significantly associated with an increase in GFR were %TWL 0.12 (IC 95% 0.006–0.23, *p* = 0.038) and baseline GFR 0.69 (IC 95% 0.63–0.75, *p* < 0.001), while GFR worsened by an average of 0.2 mL/min/1.73 m^2^ per year of age. Although GFR increased more in patients who underwent MBS than in those who received GLP‐1 agonist therapy, the difference was not statistically significant (*p* = 0.132). No association was found between the increase in GFR and the type of intervention used or the presence of diabetes (*p* = 0.118) (Table 4).

**TABLE 4:** Multivariate analysis using generalized estimating equation of factors associated with changes in GFR over the time.

	GEE analysis		
Covariate	Coefficient	95% CI	*p*‐value
Time (months)^a^	0.32	(0.13–0.52)	<0.001
Bariatric surgery^b^	2.69	(−0.81–6.2)	0.132
GFR baseline	0.69	(0.63–0.75)	<0.001
Age	−0.20	(−0.28–−0.11)	<0.001
%TWL	0.12	(0.006–0.23)	0.038
Diabetes	−1.54	(−3.4–0.39)	0.118

Abbreviations: %TWL, percentage of total weight loss; CI, confidence interval; GEE, Generalized estimating equation; GFR, glomerular filtration rate calculated by CKD‐EPI 2021.

^a^ 12 months follow‐up.

^b^ MBS compared with GLP‐1 agonist.

## 4 DISCUSSION

This study evaluated the longitudinal changes in GFR in obese patients with BMI ≥ 35 kg/m2 treated with liraglutide 3.0 mg or undergoing MBS. These results show a significant improvement in GFR at 12 months in both groups, regardless of history of T2D. Notably, these changes were primarily associated with patients' baseline renal function and %TWL, regardless of the management strategy used (surgical vs. pharmacological).

The findings documented in this study are consistent with those observed in various studies of MBS, where patients have been shown to improve their GFR after surgery. This is evident in the meta‐analysis conducted by S. C. Bilha et al, in which 42% of patients with an initial GFR below 90 mL/min improved beyond this threshold after surgery. Even in those who did not reach the goal, the average GFR improved from 68.9 to 81.6 mL/min.^16^ This phenomenon may be attributed to weight loss, which is associated with a reduction in adipose tissue, resulting in a decrease in proinflammatory adipokines, which are associated with renal injury^17^; this highlights the importance of significant weight loss in the early stages of CKD.

Recently, an exploratory analysis of SURPASS‐4 reported the renal outcomes of tirzepatide, a dual GLP‐1/GIP analog, compared with insulin glargine. The use of tirzepatide was associated with weight loss ranging from 8.1% to 13% from baseline, slower decline in GFR, and reduced albuminuria.^9^ In addition, a significant reduction in the occurrence of the composite renal outcome (≥40% decline in GFR from baseline, end‐stage renal disease, death from renal failure, or new‐onset macroalbuminuria) was observed, suggesting that significant weight loss is one of the mechanisms involved in reducing renal outcomes.^18^ Perkovic et al. showed in patients with T2D and CKD that semaglutide at a dose of 1.0 mg once weekly significantly reduced the risk of significantly reduced the risk of major renal events (initiation of long‐term dialysis, kidney transplantation, or a decrease in GFR to <15 mL/minute) by 24%.^19^ In addition, with a baseline GFR of 47.0 ± 15.2 mL/min/1.73 m^2^, annual renal function decline was slowed by a mean of 1.16 mL/min/1.73 m^2^(19).

The present study suggests that the use of liraglutide at a dose of 3 mg/day for the treatment of obesity maintains a stable GFR in the early stages of CKD and may even improve it, regardless of a history of T2D, likely due to significant weight loss. In trials of GLP‐1 agonists, meta‐analyses suggest that the renal benefit of these drugs is primarily due to a reduction in albuminuria, with no significant effect on GFR. However, it is important to note that these studies were conducted in patients with T2D and at doses lower than those used for obesity management.^20^ Two major studies support the use of GLP‐1 agonists in the management and treatment of obesity: the SCALE study with liraglutide at a dose of 3 mg/day^21^ and the STEP study with semaglutide at a dose of 2.4 mg/week, which demonstrated clear benefits in weight loss in obese patients, with weight loss ranging from 8% to 20%.^22^ However, the effect of weight loss on renal function was not reported. The recently published SELECT study, which enrolled patients with a BMI of 27 kg/m^2^ with pre‐existing major cardiovascular disease but without diabetes, compared subcutaneous semaglutide 2.4 mg weekly with placebo and showed a 20% reduction in the combined risk of any cardiovascular death, non‐fatal myocardial infarction or non‐fatal stroke.^23^ However, there was no difference in the composite end point of nephropathy.

At this time, there are no known randomized clinical trials that have evaluated the effect of GLP‐1 agonists on renal function for weight management in obese patients. Therefore, this study is the first to specifically evaluate this patient population and compare them with those undergoing MBS. Limitations of this study include retrospective data collection from an obesity clinic registry, making it impossible to retrieve lost data. To minimize this impact, missing data were individually cross‐referenced in the institution's medical records. Of note, the GLP‐1 agonist‐only group was small compared to the obese patient cohort, potentially limiting the precision of the GLP‐1 agonist effect estimate. Additional studies with larger populations are needed to validate these results.

## 5 CONCLUSION

The results of this study suggest that changes in GFR, and specifically the potential increase in GFR, are directly related to the magnitude of weight loss and baseline GFR and not to the type of therapy employed or the presence of diabetes.

## CONFLICT OF INTEREST STATEMENT

Diana Henao has received speaker fees from Novo Nordisk, Sanofi and Abbott. No other potential conflicts of interest have been reported.
